# Reversed Abdominoplasty in Aggressive Locally Advanced Breast Cancer Defect: A Safe Option for High Recurrence Risk Patients

**DOI:** 10.7759/cureus.23230

**Published:** 2022-03-16

**Authors:** Thevarasan Ganandran, Ragnild Redit, Fatimah Mat Johar, Wan Azman Wan Sulaiman, Ahmad Sukari Halim

**Affiliations:** 1 Reconstructive Sciences Unit, Universiti Sains Malaysia, Kubang Kerian, MYS

**Keywords:** reversed abdominoplasty, oncoplastic breast surgery, chest wall repair & reconstruction, breast reconstruction, autologous breast reconstruction

## Abstract

The reverse abdominoplasty technique has uses that extend past cosmetic surgery into the field of reconstructive surgery. With a thorough understanding of the technique and modifications, this method may be used to cover extensive chest wall defects post-mastectomy in select patients. Reconstructive algorithms for locally advanced breast cancer tend to favor microsurgical techniques. However, the surgeon needs to choose the optimal reconstructive option based on the defect size, disease stage, future oncological therapeutic approach, and patients’ general condition. Patients with aggressive forms of breast cancer have higher risks of recurrence despite advances in chemotherapy. This subset of patients may be poor responders to adjuvant or neoadjuvant oncological drugs, may require postoperative radiotherapy, or be at high risk for early recurrence. In this subset of patients, we advocate the use of a reverse abdominoplasty for immediate autologous soft tissue coverage of the chest wall after breast cancer resection. It provides a robust soft tissue cover for radiotherapy and spares potential future autologous donor sites for delayed breast reconstruction while facilitating early clinical detection of recurrence. Here, we discuss a case treated with this technique and explore the surgical technique, pitfalls, and advantages of this technique with the outcome that validates decision-making.

## Introduction

Reconstructive algorithms for locally advanced breast cancer recommend advanced microsurgical techniques [1,2]. Broadly, this can be accomplished in most cases. In clinical practice, however, we tend to encounter unique subsets of patients requiring innovative decision-making to improve the outcomes of multimodal cancer treatment [3].

The reverse abdominoplasty was first introduced in 1977 by Rebello and Franco [4]. While mostly dropping out of favor for traditional abdominoplasty techniques, its use in concomitant breast augmentation surgery has provided it a role in reconstructive surgery [5]. This method provides the necessary immediate stable soft-tissue coverage required for extensive chest wall defects while negating the potential pitfalls of more complex options [6,7].

Here, we report our experience reconstructing a huge unilateral left chest wall soft-tissue defect with a reverse abdominoplasty technique following resection of recurrent left breast cancer. We highlight several key points to patient selection and key techniques to using this method.

## Case presentation

A 34-year-old woman planned for wide excision of recurrent left breast carcinoma was referred to our Reconstructive Sciences Unit for left chest wall soft tissue reconstruction. She had undergone a left mastectomy and axillary clearance five months ago after completion of neoadjuvant chemotherapy for a T2N1M1, triple-negative, invasive ductal carcinoma. She had previously declined breast reconstruction after the first surgery to avoid complications. She developed local recurrence two months after the surgery while receiving and not responding to second-line chemotherapy.

**Figure 1:**
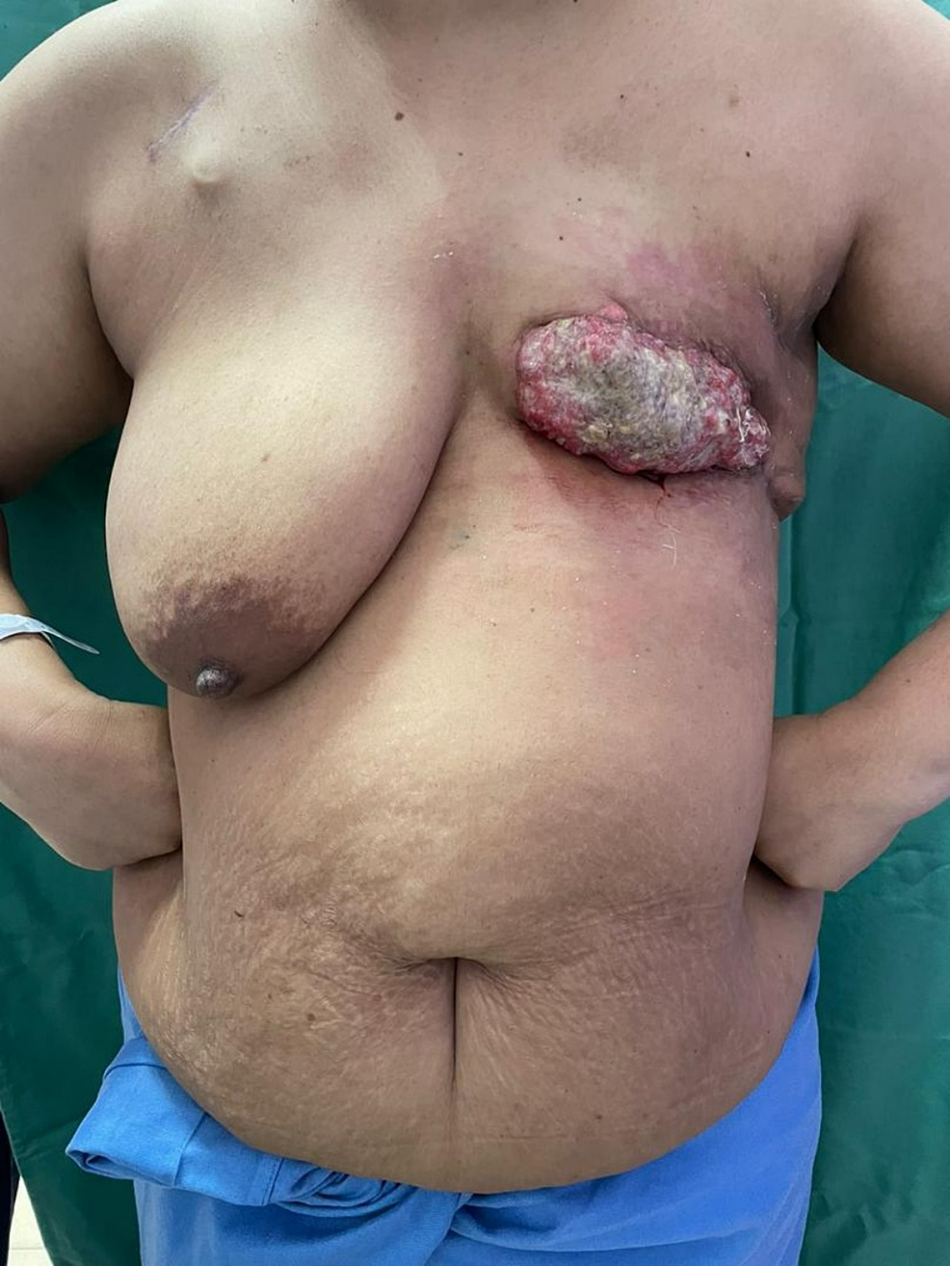
Left anterior chest wall tumor.

She had a 10 × 5 cm^2^ left chest wall fungating tumor with a palpable left axillary node (Figure 1). Contrasted computed tomography revealed pectoralis major encroachment. Her body mass index was 28.2 kg/m^2^ and she had significant upper abdominal pannus. The patient prioritized rapid recovery and low complication risk over aesthetic breast reconstruction. She was keen on a delayed reconstructive approach to facilitate the rapid transition from surgery to radiotherapy.

**Figure 2:**
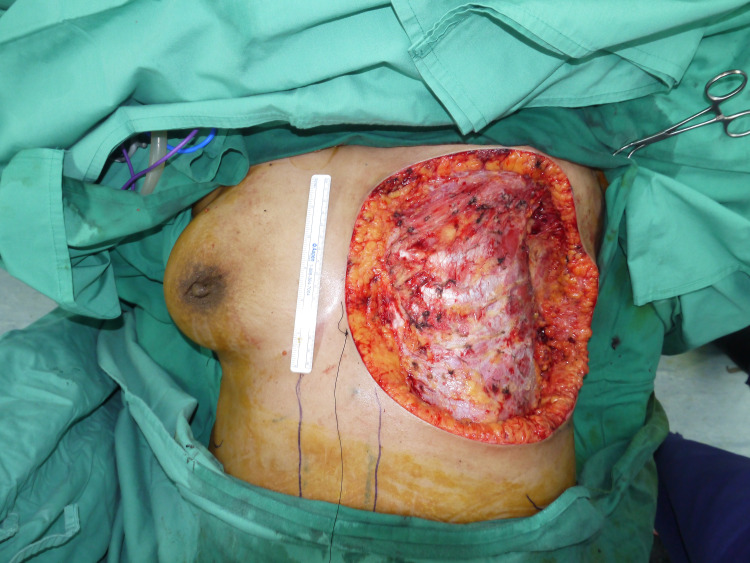
Left anterolateral chest wall defect extending inferiorly to the lower abdomen and laterally to the posterior axilla line.

The tumor was resected with adequate healthy tissue margins. This required partial excision of the pectoralis major muscle. Complete resection resulted in a 25 × 23 cm^2^ left anterolateral chest wall soft-tissue defect with exposed intercostal muscles and perichondrium (Figure 2).

**Figure 3:**
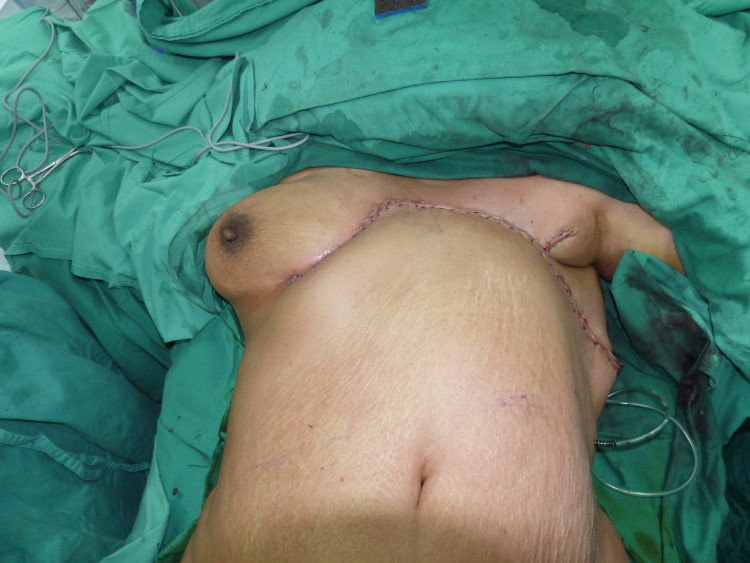
Reverse abdominoplasty rotated laterally and advanced cranially into place.

**Figure 4:**
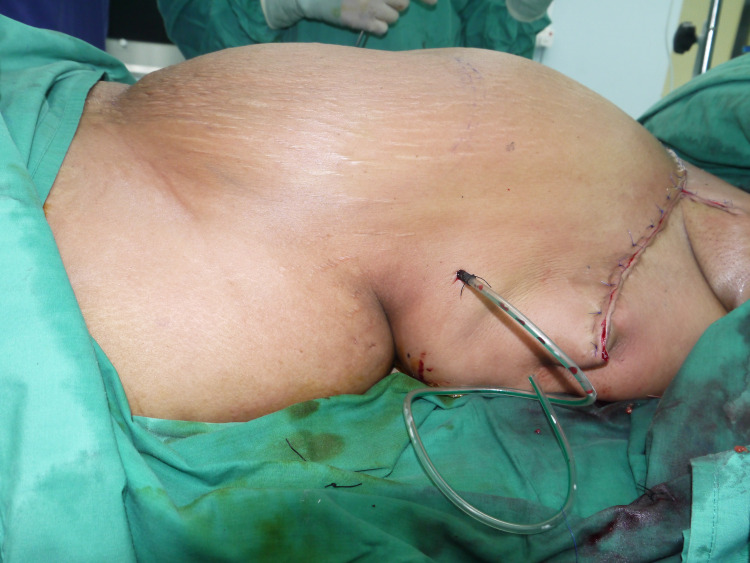
Lateral view of reverse abdominoplasty with relaxing incision extending posteriorly.

Reconstruction started with the extensive undermining of the superficial fascia cranially until the clavicle, followed by lateral undermining. Next, a “gull-wing” incision was extended from the lower border of the defect to the contralateral inframammary fold until the level of the contralateral midclavicular line. This incision facilitates a rotational element. The upper abdomen wall was raised below the level of Scarpa’s fascia. Lateral dissections were limited to the level of anterior axillary lines bilaterally to preserve the lateral intercostal blood supply. Mid-abdominal dissection was done caudally up to the supraumbilical level. The tissue was then advanced cranially and rotated onto the defect. An active drain was inserted at the recipient site. The wound was closed in layers using interrupted 2/0 polyglactin sutures for the superficial fascia and dermis. Skin closure was accomplished with vertical mattress sutures using nylon 3/0 (Figures 3, 4).

She required a minor revision surgery three weeks later for dehiscence as the superior part which is the distal-most portion raised suffered from vascular compromise. This was achieved by extending the lateral incision and rotating further medially.

**Figure 5:**
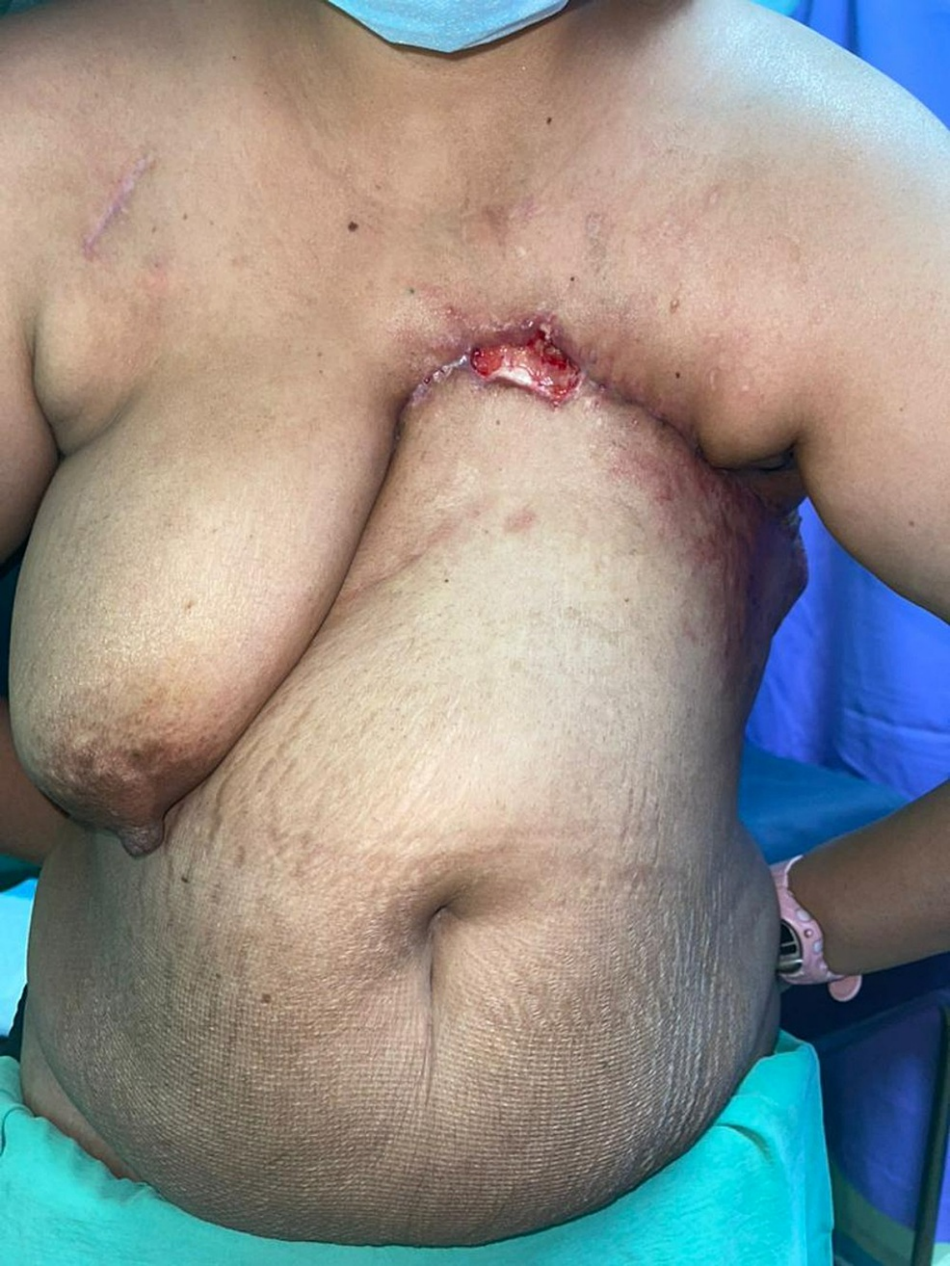
Left chest wall tumor recurrence.

She was commenced on third-line chemotherapy and planned for radiotherapy. Unfortunately, local recurrence coupled with a new contralateral breast mass was detected within six weeks of the revisional surgery (Figure 5).

## Discussion

Female breast cancer has surpassed lung cancer as the most diagnosed cancer globally (11.7%). It is the leading cause of cancer death in women (6.9%). Overall, one in four cancer cases and one in six cancer deaths in women are breast cancer-related [8]. Multimodal therapy with surgery at the forefront remains the mainstay of treatment. Advances in multimodal therapy including planning and delivery of post-mastectomy radiotherapy have significantly mitigated the risks of radiation-related complications in both subsets: autologous and implant-based reconstruction. This allows optimal dosing of radiation to selected regions with minimal complications [3].

Consequently, immediate reconstruction is generally the preferred option for the majority of cases. Published algorithms tend to side with advanced microsurgical techniques and aggressive reconstructive options for chest wall defects after tumor resection [1,2]. With huge defects, autologous reconstructive options range from locoregional flaps to free microsurgical tissue transfer. Despite arguments to both ends of the spectrum, each option carries pros and cons [1].

Surgeons need to factor in the potential catastrophic complications of flap failure, increased operating time associated with large flaps, absence of spared mastectomy skin, potential radiotherapy fat necrosis and volume loss, poor cosmesis, and suboptimal internal mammary nodes radiotherapy delivery post-reconstruction [9]. The onus remains on the surgeon to appropriately select the optimal reconstructive option based on defect size, disease stage, and patient condition. This leaves room for a subset of high-risk patients who would benefit from simpler, minimalistic immediate reconstruction. Because prognosis is dynamic with current advances in treatment, delayed reconstruction with either autologous or implant-based methods can be considered later.

A modified reverse abdominoplasty technique was chosen to reconstruct the large left anterolateral chest wall defect as a simple immediate method that additionally spares the lower abdominal tissue for potential future need. The cosmetic reverse abdominoplasty with sub-mammary incision for upper abdominal pannus was first described by Rebello and Franco in 1972 [4]. Further work by Lockwood to describe the superficial fascial planes and blood supply on the trunk provided a clever understanding of abdominoplasty anatomy [10].

By raising the upper abdominal wall, dissected below the Scarpa’s fascia, enough tissue laxity can be engaged to advance and cover chest wall defects. Blood supply comes from a robust intricate network of perforators based on the deep and superficial inferior epigastric arteries, superficial circumflex iliacs, and lumbar arteries [11].

The reverse abdominoplasty is not new to breast reconstruction. Large central chest defects resulting from bilateral mastectomies have been reconstructed with advancement methods [6,7]. However, the technique requires refinement when dealing with large unilateral defects.

We choose to dissect caudally until just superior to the umbilicus just stopping short of sacrificing vital periumbilical perforators from the epigastric system. Superior epigastric artery perforators are sacrificed along the way. Dissection is limited to anterior axillary lines bilaterally. By utilizing a “gull-wing” incision at the contralateral inframammary crease, a rotational arc can be added to the flap. Dissection at the inframammary crease should proceed inferiorly away from the breast to not completely violate the structural integrity of the inframammary fold. This incision should ideally be closed by anchoring the new inframammary fold to thoracic fascia and subsequent closure in layers to recreate the inframammary crease. In event of insufficient laxity, further undermining can be safely done beyond the umbilicus up to the suprapubic crease. Dissection of the umbilical stalk would be necessary in this case [6,12]. For such large defects, patient selection is key. The main requirement to successfully reconstruct huge defects safely is the availability of an upper abdominal pannus to be advanced. This negates the need for extensive caudal dissection. Surgeons have used this method for augmentation mammaplasty and noted that with an adequately sized pannus, 300 cc of flap volume can be harvested to reconstruct a single breast [5].

Distal necrosis and dehiscence is a potential risk. The tension on wound margins can be reduced by applying tensioned reversed abdominoplasty techniques using lines of continuous progressive tensioned sutures to fix the Scarpa’s fascia to the deep fascia during inset [13]. In the event of necrosis or possible dehiscence, the tissue can be successfully re-advanced by extending the incision laterally to provide further laxity [12].

Inherent limitations to choosing this option and recommendations to improve outcomes are as followed. The size of the upper abdominal pannus required should be proportionate to the defect. Attempting to close a huge defect with this method on a slim patient may be futile or result in a high-tension closure. This leads to potential complications such as wound dehiscence. Progressive tensioned closure and extending incisions to incorporate more rotational components into the flap reduce this risk. Patients would require bed rest to negate the effects of gravity on closure.

Scars need to be evaluated carefully before selecting this method. There is a potential of requiring extensive elevation to increase the arc of rotation leading to the need to rely upon random pattern perfusion and perfusion across midline from contralateral vasculature. Because this method relies heavily on inferolateral blood supply, midline laparotomy scars are absolute contraindications.

This is not a breast reconstruction, and, as such, patients should be made aware that delaying the breast reconstruction with this method may limit future potential options of implant-based reconstruction. Accounting for the anticipated postoperative radiotherapy, the resultant fibrosis may be a deterrent for future implant-expander approach. The reconstructive surgeon would be limited to autologous flaps should breast reconstruction be desired.

As in our patient, locoregional recurrence is always a threat with aggressive forms of breast carcinoma that are chemotherapy-resistant. By sparing valuable periumbilical perforators, we do not burn the bridge to a future possible use of abdominal-based flaps should the need arise.

## Conclusions

Reverse abdominoplasty is not a standard reconstructive approach for mastectomy patients. However, in a subset of patients who require a stable soft-tissue coverage of chest wall defects when the risk of recurrence may be higher, this method presents a salient option. Rapid surgery, minimized preoperative workup, reduced complications, easy troubleshooting, and sparing of potential lower abdominal flap donor integrity for future reconstruction are several key advantages of this approach. We advocate that surgeons carefully select the candidates for this reconstructive method to ensure good outcomes.
